# Resolution of Roemheld Syndrome After Hiatal Hernia Repair and LINX Placement: Case Review

**DOI:** 10.7759/cureus.37429

**Published:** 2023-04-11

**Authors:** Madison J Noom, Alden Dunham, Christopher G DuCoin

**Affiliations:** 1 Surgery, University of South Florida Morsani College of Medicine, Tampa, USA

**Keywords:** gerd, linx magnetic sphincter augmentation, hiatal hernia, gastrocardiac syndrome, roemheld syndrome

## Abstract

Roemheld syndrome, also known as gastrocardiac syndrome, was first studied as a relationship between gastrointestinal and cardiovascular symptoms through the vagus nerve. Several hypotheses have attempted to explain the pathophysiology of Roemheld syndrome, but the underlying process remains unclear. We present a clinically diagnosed case of Roemheld syndrome in a patient with a hiatal hernia whose gastrointestinal and cardiac symptoms were successfully treated with robotic assisted hernia repair, esophagogastroduodenoscopy (EGD), and LINX magnetic sphincter augmentation.

Our case is a 60-year-old male with a history of esophageal stricture and hiatal hernia who presented with complaints of gastroesophageal reflux disease (GERD) and related arrhythmias for five years. The patient did not have a history of cardiovascular disease other than hypertension. The cause of the hypertension was assumed to be primary, as workup for possible pheochromocytoma was negative. Cardiac work-up revealed arrhythmias that were characterized as supraventricular tachycardia with intermittent pre-ventricular contractions (PVC); however, testing was unable to determine a cause for the arrhythmias. High-resolution manometry showed low pressure in the lower esophageal sphincter with normal esophageal motility. Further evaluation included a 96-hour Bravo test and DeMeester score of 31 was recorded, confirming mild GERD; however, EGD was unremarkable. Surgeons elected to perform a robotic assisted hiatal hernia repair, EGD, and magnetic sphincter augmentation. Four months following surgery, the patient denied symptoms of GERD or episodes of palpitation and subsequently weaned off proton pump inhibitors with continual lack of symptoms.

GERD is a common complaint among the primary care setting; however, ventricular dysrhythmias among this population and a clinical diagnosis of Roemheld syndrome is unique. One hypothesis may be that protrusion of the stomach into the chest cavity may exacerbate current reflux, and the anatomical relationship between a herniated fundus and anterior vagal nerve may cause direct physical stimulation that is a more potent risk factor for the development of arrythmias. However, Roemheld Syndrome is a unique diagnosis, and the pathophysiology is still yet to be understood.

## Introduction

The association between gastrointestinal symptoms and cardiac arrhythmias was first observed by Ludwig Roemheld who described arrhythmia symptoms secondary to foregut stimulation [1]. Since this observation, several hypotheses have attempted to explain the pathophysiology of Roemheld Syndrome, but the underlying process remains unclear. The most prominent explanations include autonomic imbalance associated with reflux-induced vagus nerve stimulation or local inflammation of the left atrium triggered by the esophageal reflux due to the close anatomical association between the left atrium and esophagus. Inflammation in the esophagus caused by gastroesophageal reflux disease (GERD) prompts the release of inflammatory cytokines such as IL1β, IL6, and C-reactive protein [1]. Release of these mediators adjacent to the left atrium may cause direct effects on the heart, causing inflammation and subsequent supraventricular arrhythmias. C-reactive protein has been especially linked with atrial fibrillation (AF), and therefore may play an important role in the pathogenesis of Roemheld Syndrome [2]. Despite the high prevalence of GERD and cardiac arrhythmias, Roemheld Syndrome is rare and reports in the literature are sparse. Epidemiologic analyses of the association between GERD and AF have produced mixed results, in which some studies have found an increased risk for AF in GERD patients and others have found no such correlation [3-7]. The association between GERD and other supravetricular arrhythmias or ventricular arrhythmias, such as pre-ventricular contractions (PVC), are also not well-understood. Here, we present a clinically diagnosed case of Roemheld Syndrome in a patient with a hiatal hernia whose gastrointestinal and cardiac symptoms were successfully treated with robotic assisted hernia repair, esophagogastroduodenoscopy (EGD), and LINX magnetic sphincter augmentation.

## Case presentation

A 60-year-old male with a history of esophageal stricture, reflux esophagitis, and hiatal hernia presented to the clinic with complaints of GERD and related arrhythmias for the previous five years. Shortly after being diagnosed with GERD and reflux esophagitis, the patient subsequently began experiencing new episodes of palpitations and tachycardia, as well as synchronous episodes of hypertensive urgency. Workup for a suspected pheochromocytoma was conducted but results were found to be negative. Before these symptoms began, the patient had no previous cardiac history. He denies use of tobacco products, illicit drugs, and does not currently use alcohol. Prior medication use includes tamsulosin for nephrolithiasis two years after the start of gastro-esophageal and cardiac symptoms. At the time of clinic presentation medications included oral omeprazole, as well as metoprolol, amlodipine, and losartan for blood pressure control and prevention of episodes of hypertensive urgency. Magnesium levels were annually checked during the prior two years and were found to be within normal limits (2.2 and 2.1 mg/dL).

Cardiac work-up with CT angiography revealed normal cardiac chamber morphology, an ejection fraction of 61%, and moderate plaque burden in the right coronary artery. Exercise stress test results were unremarkable. The arrhythmias were characterized as supraventricular tachycardia with intermittent PVC.

**Figure 1:**
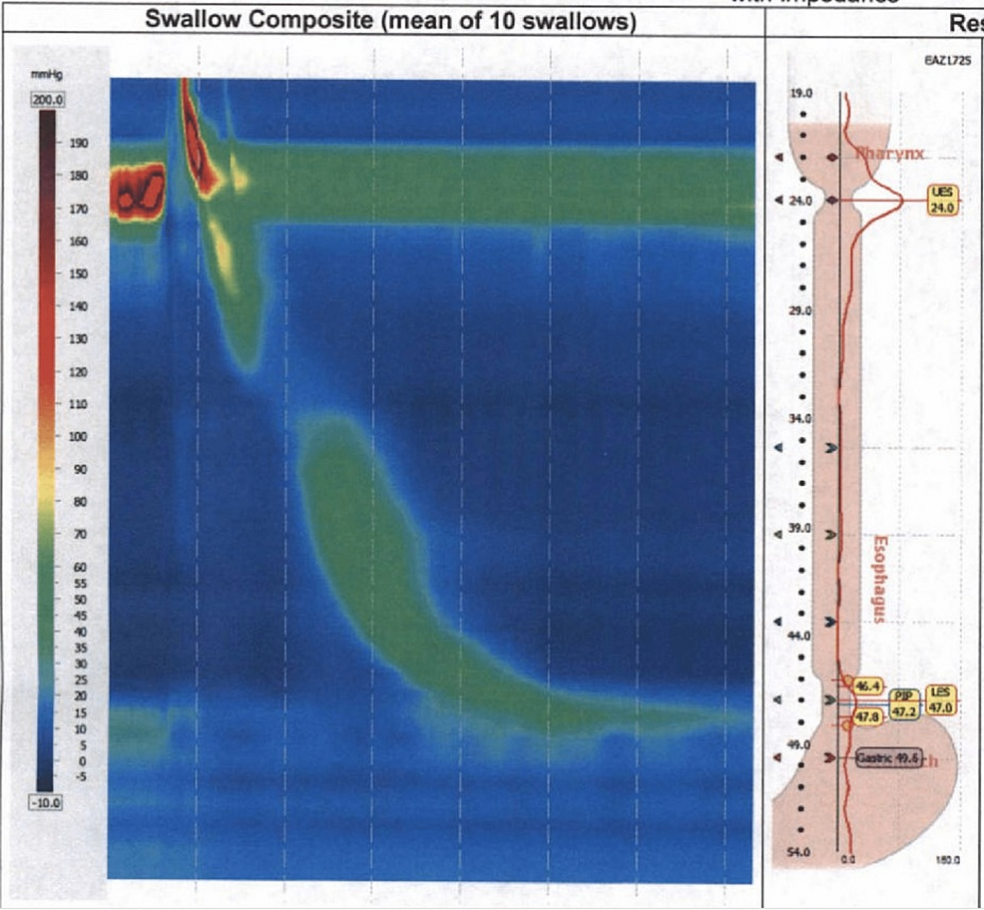
High-resolution manometry LES: Lower esophageal sphincter; PIP: Pressure inversion point

The patient later returned for a follow up esophageal motility study; however, on the morning of the imaging, a bolus became stuck in his esophagus, which produced subsequent abdominal distention and tachycardia. The tachycardia resolved once the bolus was removed with Valsalva and burping. Later that afternoon, high-resolution manometry (Figure 1, Table 1, Table 2) showed low pressure in the lower esophageal sphincter with normal esophageal motility. 

 

**Table 1:** Reflux monitoring—acid exposure summary

Acid Exposure Summary	Total	Normal	Upright	Supine
Acid exposure time (%)	11.1	<6.0	16.4	0.0
Longest result (min)	24.4	<16.0	24.4	0.2
DeMeester Score	31.0	<14.7		

**Table 2:** Reflux summary—symptom association summary

Symptom Association Summary	Heartburn	Chest Pain
Number of occurrences	6	2
Symptom index for reflux (SI)	16.7	0.0
Symptom association probability (SAP)*	59.7	73.4

**Figure 2:**
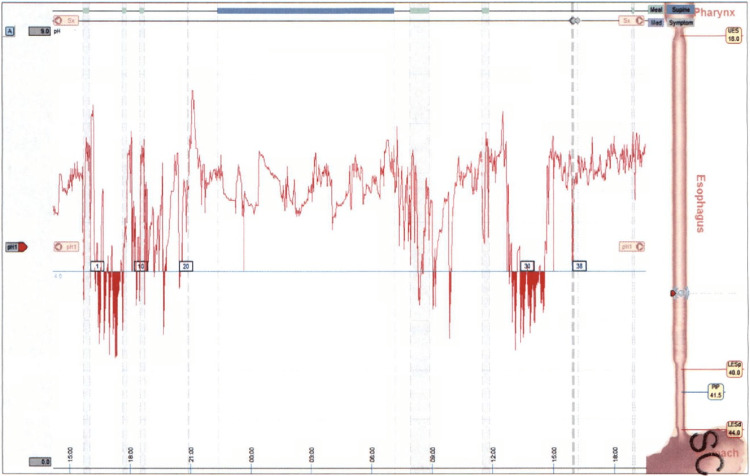
Charted 96-hour Bravo test results UES: Upper esophageal sphincter; LES: Lower esophageal sphincter; PIP: Pressure inversion point

After the procedure, the patient experienced a subsequent 48 hours of continuous cardiac arrhythmias. Further evaluation included a 96-hour Bravo test (Figure 2, Table 3) that was to be performed two weeks after the stoppage of omeprazole. A DeMeester score of 31 was recorded, confirming mild GERD; however, EGD was unremarkable.

**Table 3:** 96-hour Bravo test results LES: Lower esophageal sphincter

Esophageal Motility		Measured	Normal
Number of swallows evaluated		10	
Chicago classification			
	% Failed	0	
	% Weak	0	
	% Panesophageal pressurization	0	
	% Premature contraction	0	
	% Intact	0	
Additional high-resolution parameters			
	Distal latency	6.6	
	Distal contractile integral (Mean)	883.4 mmHg	500-5,000 mmHg
	Contractile front velocity	3.4 cm/s	< 9.00
Evaluated @ 3.0 & 7.0 cm above LES			
	Mean wave amplitude	61.5 mmHg	43-152 mmHg
Impedance analysis			
	Incomplete bolus clearance	0%	
	Bolus transit tem	2.6 sec	

The patient was subsequently started on a greater dose of omeprazole which gave almost complete GERD symptom relief, as well as relief of arrhythmias. With the goal of complete symptom relief and weaning off proton pump inhibitors, surgeons elected to perform a robotic assisted hiatal hernia repair, EGD, and magnetic sphincter augmentation. Magnesium levels were measured before surgery, and were once again found to be within normal limits (1.7 mg/dL). Of note, during the procedure a substantial amount of periesophageal inflammation was encountered.

Shortly after surgery, the patient had an episode of supraventricular tachycardia with new ST-segment deviation and heart rate ranging from 110-130 bpm. Symptoms were relieved with intravenous metoprolol and the patient subsequently began oral amlodipine. At first follow up appointment two weeks post-operatively, the ECG showed a regular rate and rhythm and the patient began weaning off of oral proton pump inhibitors over the subsequent two weeks. Throughout the post-operative period, the patient was not treated by a cardiologist and was weaned off all rate-control medications shortly after surgery. At the next follow-up appointment, four months after surgery and discontinuation of all proton-pump inhibitors and rate-control medications, the patient denied symptoms of GERD or episodes of palpitation. These symptoms continued to be denied at the third follow-up appointment ten months post-operatively. During this time, the patient also did not have any episodes of hypertensive urgency.

## Discussion

GERD and cardiac arrhythmias are both highly prevalent conditions affecting a significant proportion of the United States population. Despite this prevalence, the epidemiological link between the GERD and cardiac arrhythmia is unclear, and the clinical diagnosis of Roemheld Syndrome is unique [8,9]. Only minimal cases have been described and current literature lacks a study specifically describing the incidence of this diagnosis. This poses the question of what differences in these patients mediate their gastrocardiac symptoms.

One hypothesis is that the cause of the periesophageal inflammation needed to stimulate the vagus nerve is a two-step process that requires both inflammatory mediators released from reflex esophagitis, as well as the altered blood supply to the stomach due to the hiatal hernia [1]. In the few cases of Roemheld syndrome described, patients had both GERD and an uncorrected hiatal hernia [10]. Protrusion of the stomach into the chest cavity may exacerbate current reflux and physically aggravate the vagus nerve and/or the heart itself, increasing the risk of dysrhythmia.

Although current literature does not include investigations into association between gastrointestinal diseases and ventricular arrhythmias, such as PVCs like what was seen in our patient, studies have shown that conditions like GERD and hiatal hernias may have a correlation with other arrhythmias. Roy et. al found that there was a significant increase in AF among both men and women who also had a hiatal hernia. This correlation was most significant in a younger population (<55 years old) but did exist in all age groups [11]. As discussed earlier, current literature has not consistently shown GERD by itself to be a risk factor for development of AF [3-7]. This may indicate that GERD itself does not cause enough peri-esophageal inflammation to lead to gastro-cardiac syndrome, and additional co-morbidities, such as a hiatal hernia, must be present to affect the heart. Due to the anatomical relationship between a herniated fundus and anterior vagal nerve, it may be this direct physical stimulation that is a more potent risk factor for the development of arrhythmias.

Diagnosis of Roemheld Syndrome should be considered in patients with anti-arrhythmic resistant supraventricular arrhythmias and a significant history of GERD, especially those who also have a hiatal hernia. Although the patient presented here did not endorse classic timing of symptoms, signs that may point to gastrocardiac phenomena are palpitations, dizziness, or shortness of breath in the presence of GERD symptoms or meal consumption. Patients who present with these symptoms who have not been diagnosed with an arrhythmia may need to be considered for periodic electrocardiograms to detect presence of AF or other types of cardiac arrhythmia. AF may occur asymptomatically for prolonged periods of time before patients begin to feel palpitations, fatigue, or shortness of breath, and, therefore, can go undetected for prolonged periods of time. Screening this population who may be at increased risk for AF or other cardiac arrhythmias may be used to prevent stroke due to hemostasis in the left atrium or left ventricle. The general treatment for gastrocardiac syndrome is to treat the underlying gastrointestinal pathology in hopes of preventing further vagal nerve and/or left atrial irritation, thus preventing cardiac symptoms. This includes medical management of GERD and hiatal hernia repair but should be tailored to each individual patient. These patients should also be followed by cardiology in order to manage their cardiac arrhythmia while gastrointestinal symptoms are continuing to resolve.

## Conclusions

Roemheld Syndrome is a unique diagnosis for which the pathophysiology is still yet to be understood. Literature suggesting an increase in AF among patients with a hiatal hernia support that it is stimulation of the vagus nerve that shortens effective atrial refractoriness, leading to arrhythmia. Other reports support the role of local inflammation via release of inflammatory mediators in this connection between gastroesophageal and cardiac pathology. Regardless of the mechanism, it is unclear why some GERD patients develop AF and only a very small population develop ventricular arrhythmias. Additional studies comparing risk factors among patients with a hiatal hernia who develop AF and those who develop ventricular arrythmias may be warranted. However, sample size may be limited, as the population of patients with a diagnosis of Roemheld Syndrome is yet to be assessed. Awareness of Roemheld Syndrome may help providers recognize gastrocardiac symptoms in their patients and when possible, use the treatments for gastrointestinal symptoms to prevent a need for use of anticoagulation or antiarrhythmic treatment for AF control. 
